# Mutation of *Rubie*, a Novel Long Non-Coding RNA Located Upstream of *Bmp4*, Causes Vestibular Malformation in Mice

**DOI:** 10.1371/journal.pone.0029495

**Published:** 2012-01-12

**Authors:** Kristina A. Roberts, Victoria E. Abraira, Andrew F. Tucker, Lisa V. Goodrich, Nancy C. Andrews

**Affiliations:** 1 Department of Pediatrics and Department of Pharmacology and Cancer Biology, Duke University, Durham, North Carolina, United States of America; 2 Graduate Program in Biological and Biomedical Sciences, Harvard Medical School, Boston, Massachusetts, United States of America; 3 Department of Neurobiology, Harvard Medical School, Boston, Massachusetts, United States of America; Stanford University School of Medicine, United States of America

## Abstract

**Background:**

The vestibular apparatus of the vertebrate inner ear uses three fluid-filled semicircular canals to sense angular acceleration of the head. Malformation of these canals disrupts the sense of balance and frequently causes circling behavior in mice. The *Epistatic circler* (*Ecl*) is a complex mutant derived from wildtype SWR/J and C57L/J mice. *Ecl* circling has been shown to result from the epistatic interaction of an SWR-derived locus on chromosome 14 and a C57L-derived locus on chromosome 4, but the causative genes have not been previously identified.

**Methodology/Principal Findings:**

We developed a mouse chromosome substitution strain (CSS-14) that carries an SWR/J chromosome 14 on a C57BL/10J genetic background and, like *Ecl*, exhibits circling behavior due to lateral semicircular canal malformation. We utilized CSS-14 to identify the chromosome 14 *Ecl* gene by positional cloning. Our candidate interval is located upstream of bone morphogenetic protein 4 (*Bmp4*) and contains an inner ear-specific, long non-coding RNA that we have designated *Rubie* (RNA upstream of *Bmp4* expressed in inner ear). *Rubie* is spliced and polyadenylated, and is expressed in developing semicircular canals. However, we discovered that the SWR/J allele of *Rubie* is disrupted by an intronic endogenous retrovirus that causes aberrant splicing and premature polyadenylation of the transcript. *Rubie* lies in the conserved gene desert upstream of *Bmp4*, within a region previously shown to be important for inner ear expression of *Bmp4*. We found that the expression patterns of *Bmp4* and *Rubie* are nearly identical in developing inner ears.

**Conclusions/Significance:**

Based on these results and previous studies showing that *Bmp4* is essential for proper vestibular development, we propose that *Rubie* is the gene mutated in *Ecl* mice, that it is involved in regulating inner ear expression of *Bmp4*, and that aberrant *Bmp4* expression contributes to the *Ecl* phenotype.

## Introduction

The vestibular apparatus of the vertebrate inner ear is an intricate structure responsible for sensing motion and position of the head. Within the vestibular system, angular head movements are detected by three fluid-filled semicircular canals oriented in nearly orthogonal planes. Fluid movement is generated by changes in head position and detected by complex sensory structures, known as cristae ampullares, located at the base of each canal. This precise inner ear architecture is crucial for proper vestibular function and even small structural changes can profoundly impact the ability to detect and transmit sensory information. Vestibular abnormalities can lead to debilitating dizziness and imbalance in humans [1], [2] and frequently cause circling behavior in mice [3], [4]. Inner ear morphogenesis is a highly regulated process that depends upon precise spatiotemporal patterns of gene expression to generate three-dimensional structures [5], [6]. Identifying these genes and elucidating their functions is key to better understanding this intricate process, and mouse models of vestibular dysfunction have proven remarkably useful in this regard [3], [4].

The *Epistatic circler* (*Ecl*) is a complex mouse mutant originally discovered in a multi-generation intercross population derived from wildtype SWR/J and C57L/J mice [7]. In *Ecl* mice, circling and hyperactivity result from bilateral malformation of the lateral semicircular canal (LSC), while the anterior and posterior canals are unaffected [8]. Circling is caused by the epistatic interaction of a recessive, SWR-derived gene on chromosome 14 and a dominant, C57L-derived gene on chromosome 4. Additional C57L alleles at modifier loci on chromosomes 3 and 13 increase circling risk [9]. While the broad chromosomal locations of these four *Ecl* loci have been defined, none of the underlying genes have yet been identified.

Here we describe the development of a mouse chromosome substitution strain that genetically resembles *Ecl*, and exhibits similar behavioral and structural abnormalities. We utilized this new mouse strain to identify a likely causative gene at the chromosome 14 *Ecl* locus by positional cloning. Our candidate interval lies upstream of bone morphogenetic protein 4 (*Bmp4*), a gene known to be essential for proper vestibular development [10], [11], [12], [13], [14]. While *Bmp4* is excluded as an *Ecl* candidate, we discovered a long, non-coding RNA (ncRNA) within our interval that is co-expressed with *Bmp4* in developing semicircular canals. We demonstrate that expression of this novel ncRNA is disrupted by an SWR-specific endogenous retrovirus and propose a role for it in regulating *Bmp4* expression.

## Results

### Generation of a Chromosome Substitution Strain that Exhibits Circling Behavior

We developed a mouse chromosome substitution strain carrying an SWR/J (SWR) chromosome 14 on a C57BL/10J (B10) genetic background (C57BL/10J-Chr14^SWR/J^, referred to here as CSS-14). While heterosomic CSS-14 mice (Chr14^B10/SWR^ on a B10/B10 background) showed no behavioral abnormalities, a subset of their intercross progeny displayed hyperactivity and bidirectional circling that was evident before weaning and persisted throughout life. We recognized that the strain combination of CSS-14 resembled that of *Ecl*, and hypothesized that the same epistatic interaction was responsible for circling in both mutants. All CSS-14 mice are homozygous for C57-derived alleles at *Ecl* loci on chromosomes 3, 4, and 13, and genotyping of intercross progeny confirmed that CSS-14 circlers always inherit two SWR alleles at the recessive *Ecl* locus on chromosome 14 (Chr14^SWR/SWR^). This inheritance pattern strongly suggests that we unintentionally recapitulated *Ecl* in the construction of CSS-14. As previously hypothesized for *Ecl*, CSS-14 circling is not fully penetrant [7], [9]; we observed circling in 54% of Chr14^SWR/SWR^ mice.

### CSS-14 Circlers Exhibit Malformation of the Lateral Semicircular Canal

**Figure 1:**
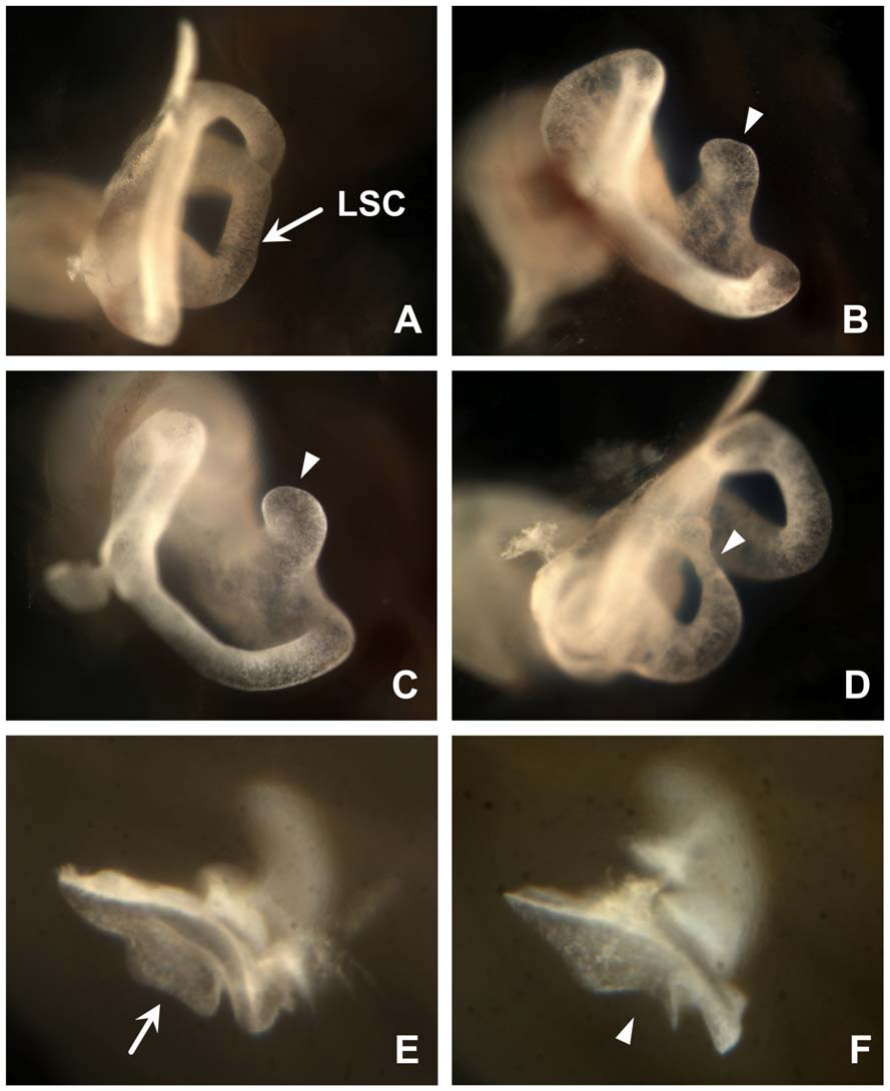
CSS-14 circlers exhibit malformation of the lateral semicircular canal. (A) Inner ear morphology of CSS-14 mice was assessed at P0 using a standard paintfill technique. Chr14^B10/SWR^ mice do not circle and have intact LSCs (arrow). (B–D) 50% of inner ears from Chr14^SWR/SWR^ mice exhibit truncation (B,C) or severe distortion (D) of the LSC (arrowheads), while the anterior and posterior canals are unaffected. (E) Paintfilling was also used to assess canal pouch morphology of CSS-14 embryos at E12.25. Lateral canal pouches of Chr14^B10/SWR^ embryos exhibit normal morphology (arrow). (F) Lateral pouches from E12.25 Chr14^SWR/SWR^ embryos are abnormally-shaped and lack the distal rim necessary for proper LSC formation (arrowhead).

Circling behavior can be caused by a wide range of vestibular abnormalities. In *Ecl* mice, circling results from bilateral malformation of the LSC, with defects including complete truncation, severe distortion, and narrowing of the canals [8]. To determine whether CSS-14 circlers exhibit similar structural defects, we visualized inner ears from Chr14^B10/SWR^ and Chr14^SWR/SWR^ pups at postnatal day 0 (P0) using a standard paintfill technique. Chr14^B10/SWR^ inner ears displayed normal morphology with intact LSCs (Figure 1A). In contrast, truncation or severe distortion of the LSC was observed in six of twelve Chr14^SWR/SWR^ ears (Figure 1B–D); the anterior and posterior semicircular canals, as well as the cochlea, appeared normal in all Chr14^SWR/SWR^ ears examined. These results confirm that CSS-14 and *Ecl* circling are caused by the same type of structural malformation and further support the conclusion that both mouse models result from the same genetic defect.

Semicircular canals develop from two epithelial outpocketings of the otic vesicle. Anterior and posterior canals develop from a single vertical pouch and the LSC develops from a horizontal pouch. Following fusion and resorption of epithelial tissue at the center of the pouch, a tube-shaped canal remains along its perimeter [15]. Analyzing pouch morphology during morphogenesis can provide important information about the etiology of postnatally-observed vestibular malformation. Because *Ecl* inner ears had not previously been studied during embryogenesis, we used paintfilling to visualize canal pouches of CSS-14 embryos at E12.25. Chr14^B10/SWR^ embryos exhibited normal lateral pouches (Figure 1E), while those of Chr14^SWR/SWR^ embryos frequently lacked the distal rim necessary for proper LSC formation (Figure 1F). This observation is consistent with defects in either pouch outgrowth or fusion [11], [16].

### Positional Cloning of the Chromosome 14 *Ecl* Gene

**Figure 2:**
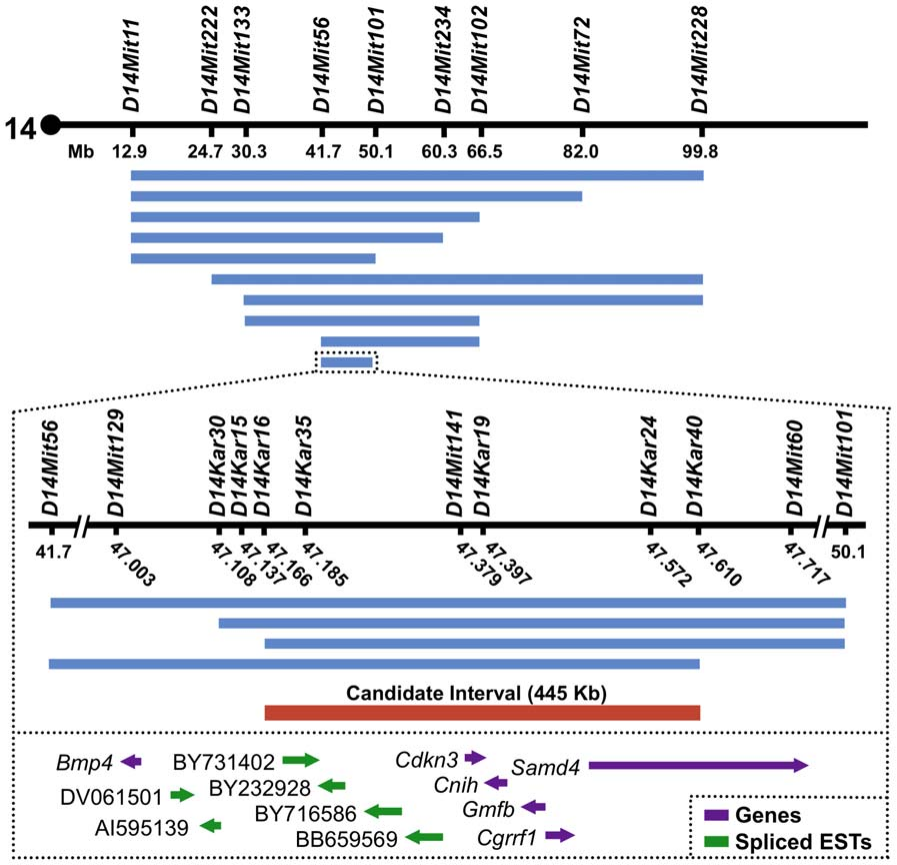
Positional cloning of the chromosome 14 *Ecl* gene. CSS-14 offspring were phenotyped as circling or non-circling and genotyped at SSLP markers across chromosome 14. Due to incomplete penetrance of the circling phenotype, only circlers were used to narrow the candidate interval. Blue bars depict regions of SWR homozygosity in CSS-14 circlers; each bar represents multiple circling mice with the same genotype. The *Ecl* candidate gene interval (red bar) was defined as the region of SWR homozygosity common to all CSS-14 circlers. This interval spans 445 Kb from D14Kar16 to D14Kar40 and includes five annotated genes and four spliced ESTs (bottom panel). The coding sequence of *Bmp4* was excluded from the candidate interval by two independent crossovers.

Next, we utilized CSS-14 mice to identify the chromosome 14 *Ecl* gene by positional cloning. Offspring of (Chr14^SWR/SWR^×Chr14^B10/SWR^) matings were phenotyped as circling or non-circling and genotyped at markers across chromosome 14. The resulting candidate gene interval, defined as the region of SWR homozygosity common to all CSS-14 circlers, spanned 445 Kb from D14Kar16 to D14Kar40 (Figure 2). This region, extending from 155 Kb to 600 Kb upstream of (telomeric to) *Bmp4*, contains five annotated genes (*Cdkn3*, *Cnih*, *Gmfb*, *Cgrrf1*, *Samd4*) and genes for four spliced ESTs (Figure 2). None of the annotated genes in this interval are known to be involved in inner ear development.

Importantly, the coding sequence of *Bmp4* was excluded from our candidate interval by two independent crossovers (Figure 2). Prior to genetic exclusion, *Bmp4* was a strong *Ecl* candidate because it is expressed in developing semicircular canals and known to play a crucial role in inner ear development [10], [11], [12], [13], [14]. In mice, homozygous deletion of *Bmp4* in the inner ear results in agenesis of all three semicircular canals [10], while heterozygous deletion causes LSC defects indistinguishable from those of CSS-14 circlers [10], [11]. With *Bmp4* excluded as a candidate, we examined the genes and spliced ESTs located within our interval more closely.

### *Rubie* is a Long, Non-Coding RNA Expressed in Developing Semicircular Canals

**Figure 3:**
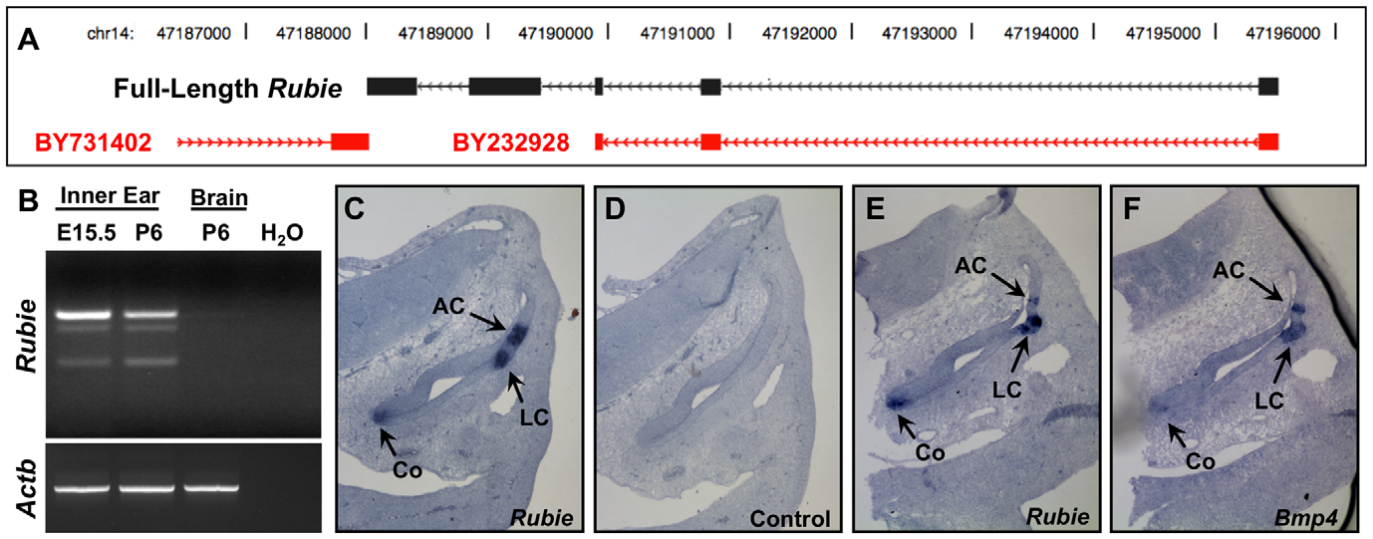
Sequence and expression analysis of *Rubie*. (A) *Rubie* (EST BY232928) sequence displayed in genomic databases is derived from a partially-sequenced RIKEN cDNA clone and includes 388 bp, divided among three exons. Resequencing of the RIKEN clone revealed a full-length insert of 1404 bp, divided among five exons. (B) Expression of *Rubie* in embryonic (E15.5) and postnatal (P6) mouse inner ears was confirmed by RT-PCR. Expression was not observed in P6 hindbrain. The bottom PCR band represents use of an alternative splice site between exons 1 and 2 of *Rubie*. (C) *Rubie* expression was detected in the three sensory cristae (anterior crista, AC; lateral crista, LC; posterior crista, not shown) and the developing cochlea (Co) of sectioned E11.5 embryos by *in situ* hybridization. (D) No signal was detected using a *Rubie* sense control probe. *Rubie* (E) and *Bmp4* (F) exhibited similar expression patterns in the developing inner ear.

One of the four ESTs in our candidate interval, BY232928, was originally identified in a RIKEN inner ear cDNA library and is not expressed in other tissues (data not shown). BY232928 lies upstream of *Bmp4*, within a genomic region previously shown to be important for *Bmp4* expression in the developing inner ear [17]. While genomic databases display 388 bp divided among three exons, our resequencing of the BY232928 RIKEN clone revealed a 1404 bp cDNA insert comprised of five exons (Figure 3A; Table S1). We refer to the gene encoding this EST and its RNA product as *Rubie* (RNA upstream of *Bmp4* expressed in inner ear). We confirmed expression of *Rubie* in embryonic (E15.5) and postnatal (P6) inner ears by RT-PCR (Figure 3B) and performed *in situ* hybridization in sectioned E11.5 embryos (Figure 3C,D). *Rubie* was detected in the three presumptive sensory cristae that give rise to semicircular canals, and at the tip of the developing cochlea. This expression pattern is strikingly similar to that of *Bmp4* (Figure 3E,F) [10], [18].

**Figure 4:**
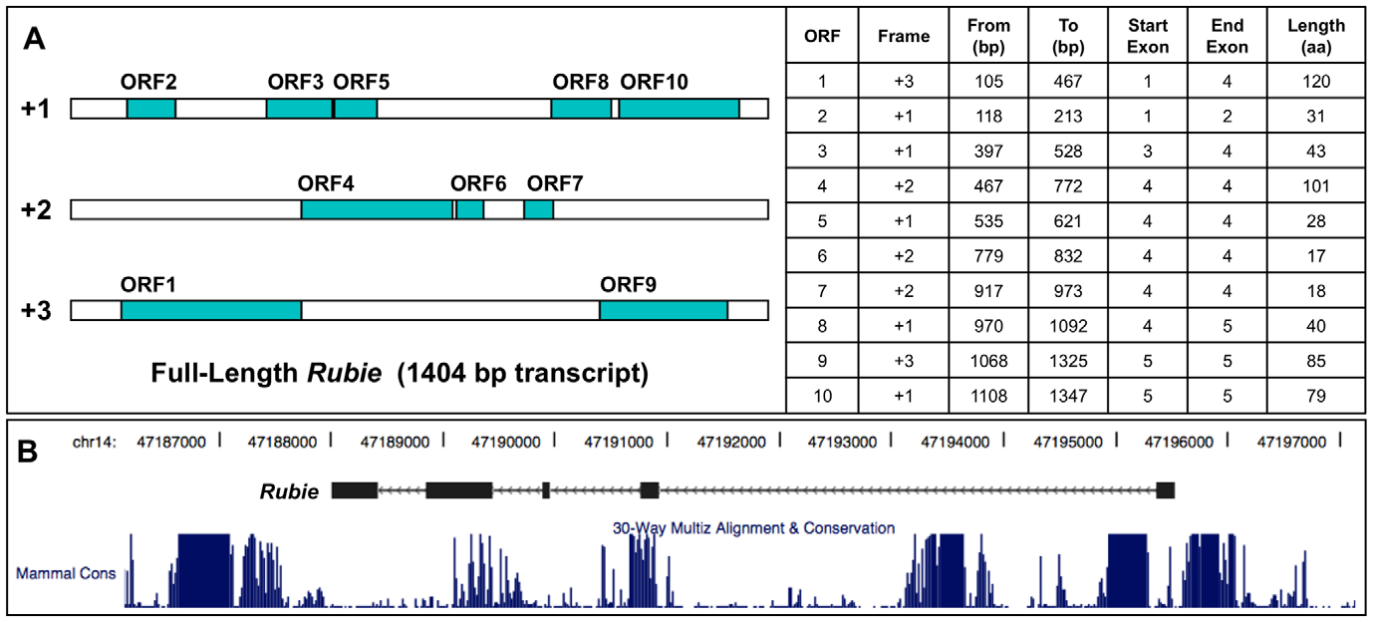
*Rubie* exhibits features of a long, non-coding RNA. (A) *Rubie* contains ten potential open reading frames longer than 50 bp. The longest (ORF1) would encode a 120 amino acid (aa) protein, but is only present in mice from the C57 lineage. Other strains have a termination codon in exon 2, which truncates the ORF at 45 aa. (B) The mammalian conservation track of the UCSC Genome Browser (http://genome.ucsc.edu/) shows that the exon sequence of *Rubie* is poorly conserved across species.

*Rubie* is a spliced, polyadenylated transcript that we believe functions as an mRNA-like, long ncRNA. Long ncRNAs are abundantly expressed in eukaryotes and recent studies suggest that many are involved in regulation of gene expression and development. However, only a small subset of these have been functionally characterized [19], [20], [21]. Long ncRNAs lack obvious predictive features and, therefore, are typically defined by their failure to exhibit protein-coding characteristics [22]. They generally contain only short (<100 amino acids) open reading frames (ORFs) and are less likely than protein-coding RNAs to be conserved across species or have significant homology to known proteins [22], [23]. *Rubie* has ten potential ORFs, the longest of which begins at the fifth ATG and could encode 120 amino acids (Figure 4A), but similar to all preceding ATGs, lacks consensus features for translational initiation. Furthermore, this ORF is only present in mouse strains of the C57 lineage; it is disrupted by a termination codon in other strains. *Rubie* exons are poorly conserved across species (Figure 4B) and BLAST analysis revealed no significant homology to known proteins. We were unable to detect a protein product by *in vitro* transcription/translation (not shown).

### An SWR-Specific Endogenous Retrovirus Disrupts *Rubie* Splicing and Expression

**Figure 5:**
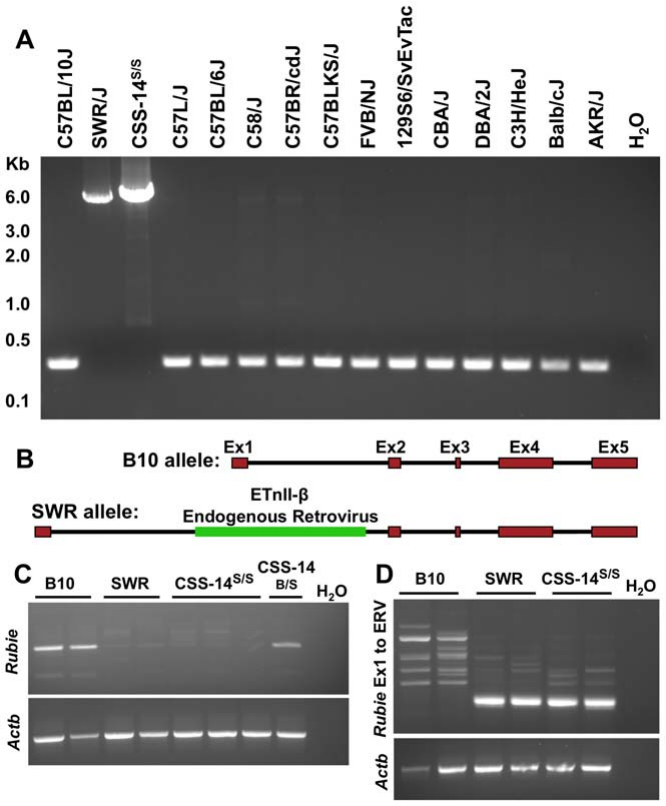
Disruption of *Rubie* by an SWR-specific endogenous retrovirus. (A) Long-range PCR spanning a previously-mapped 27 bp region of *Rubie* intron 1 (shown in Figure S2) yielded the expected 300 bp product in B10, while a larger product of approximately 6 Kb was generated in SWR mice and CSS-14 mice with the Chr14^SWR/SWR^ genotype. Sequence analysis of this larger product revealed insertion of a 5542 bp endogenous retrovirus in the SWR allele. All other inbred strains produced the shorter PCR product, indicating that this insertion is unique to SWR. (B) The B10 and SWR alleles of *Rubie* are depicted schematically. (C) *Rubie* expression in the inner ears of B10, SWR, and CSS-14 mice at P6 was assessed in cDNA using PCR primers spanning exons 1 and 2 of *Rubie*. *Rubie* expression was observed in B10 and Chr14^B10/SWR^ mice, but not in SWR or Chr14^SWR/SWR^ mice. (D) Hemi-nested PCR using forward primers in exon 1 of *Rubie* and a reverse primer in the ERV demonstrated that aberrant splicing to the ERV occurs in both SWR inbreds and CSS-14 mice.

While C57-lineage mice are frequently crossed to other inbred strains, *Ecl* has never been recapitulated by crosses that do not involve SWR. Therefore, we reasoned that the SWR allele of the causative chromosome 14 gene would likely be unique among inbred strains. We sequenced *Rubie* in SWR, B10, and thirteen additional mouse strains. Twelve single nucleotide polymorphisms distinguished SWR and B10, but none were unique to SWR (Figure S1). Sequencing of *Rubie* introns in four inbred strains (B10, SWR, 129S6/SvEvTac, and FVB/NJ) identified numerous polymorphisms, but again none were SWR-specific. However, we were consistently unable to amplify a portion of intron 1 from SWR genomic DNA, despite robust amplification in other strains (Figure S2). Using long-range PCR, we identified a large insertion in the SWR allele of *Rubie* (Figure 5A,B) attributable to a 5542 bp ETnII-β endogenous retrovirus (ERV) [24] (Figure S2). Analysis of additional inbred strains indicated that this insertion is found only in SWR (Figure 5A); even the closely-related FVB/NJ strain does not carry this ERV insertion in *Rubie*.

Intronic ETnII-β insertions have been previously shown to disrupt transcript processing in two ways: by aberrantly splicing ERV sequence into the transcript, or by coupling aberrant splicing with premature polyadenylation within the ERV [25], [26]. These effects have generally been shown to occur when the ERV is inserted in the sense orientation with respect to the affected gene, as is the case with *Rubie*. To determine whether *Rubie* processing is affected by this intronic SWR insertion, we isolated RNA from inner ears of B10, SWR and CSS-14 mice at postnatal day 6 (P6). PCR amplification spanning exons 1 and 2, 1 and 4, or 2 and 4, reproducibly failed to detect *Rubie* expression in SWR mice and CSS-14 mice with the Chr14^SWR/SWR^ genotype (Figure 5C and not shown). However, splicing of *Rubie* exon 1 to a previously-characterized ETnII-β splice acceptor [26] was detected in both SWR and CSS-14 (Figure 5D), suggesting that *Rubie* expression is disrupted by aberrant splicing coupled with premature polyadenylation. Based on these results, we conclude that the ERV insertion in intron 1 of *Rubie* significantly reduces expression of the SWR allele.

## Discussion

Based on our findings that *Rubie* expression is restricted to the inner ear, that it is expressed in the sensory cristae that give rise to the semicircular canals, that it is the only compelling candidate gene in the chromosome 14 *Ecl* candidate interval and that the insertional mutation is specific to SWR, we conclude that this mutation is likely responsible for the circling phenotype of CSS-14 and *Ecl*.

*Rubie* is a long ncRNA expressed in the cristae of the developing inner ear in a pattern identical to *Bmp4*, the transcript of a gene located less than 200 kb downstream of (centromeric to) *Rubie*. If its coding sequence had not been excluded through positional cloning, *Bmp4* would have been an ideal candidate for the chromosome 14 *Ecl* gene. The proximity of *Rubie* and *Bmp4* suggested to us that *Rubie* might be involved in the control of *Bmp4* gene expression.

*Bmp4*, like other BMPs, regulates many developmental processes and displays numerous spatiotemporal-specific expression patterns throughout development. These complex expression patterns are controlled by multiple tissue-specific, *cis*-regulatory elements, which are often located far from the coding regions and promoters of the genes they regulate [27]. BAC transgenic studies have been used to map tissue-specific regulatory elements surrounding *Bmp5*, *Gdf6*, *Bmp2*, and *Bmp4* [17], [28], [29], [30]. While global *Bmp4* deletion causes embryonic lethality, it has been suggested that genetic variation within *cis*-regulatory elements might affect tissue-specific *Bmp4* expression and result in tissue- or organ-restricted developmental defects [17].

Distant non-coding regulatory elements frequently reside within conserved gene deserts found adjacent to BMPs and other developmentally-regulated genes [27], [31]. The conserved gene desert surrounding *Bmp4* spans approximately 1 Mb, with 370 Kb lying upstream of (telomeric to) the *Bmp4* transcriptional start site. Chandler and colleagues demonstrated that an unidentified regulatory element important for inner ear expression of *Bmp4* lies in this gene desert, between 28 and 199 Kb upstream of the gene [17]. *Rubie* is an inner ear-specific, long ncRNA in that region. The expression patterns of *Rubie* and *Bmp4* are nearly identical in developing inner ears and the anatomical phenotype of mice haploinsufficient for *Bmp4* is indistinguishable from that of *Ecl* mice and CSS-14 circlers [10], [11].

Taken together, our results suggest that *Rubie* might be involved in the quantitative or temporal regulation of *Bmp4* expression in the inner ear. Perturbation of *Rubie* expression resulting from the SWR mutation might cause either a modest alteration in *Bmp4* expression, analogous to *Bmp4* heterozygosity, or abnormal timing of *Bmp4* expression. Either mechanism could disrupt the most sensitive aspect of vestibular development, the formation of the LSC. Intriguingly, there are several other tissue-restricted, spliced ESTs upstream of *Bmp4*, including BY731402 (eye), BY716586 (testis), and BB659569 (heart). We speculate that these might also be involved in tissue-specific regulation of *Bmp4* expression.

In humans, the gene desert upstream of *BMP4* has been shown to express unspliced, but no known spliced ESTs and no *Rubie*-like RNA has yet been identified. This may simply be an ascertainment issue, as there has been relatively little characterization of human EST expression during early development. Because long ncRNAs are typically not well-conserved across species, it is possible that a human long ncRNA with little sequence homology to *Rubie* may serve a similar function. Alternatively, tissue-specific regulation of *Bmp4* expression may be achieved via different mechanisms in human and mouse.

We do not yet have a model for how *Rubie* might regulate *Bmp4* expression, and previous studies of long ncRNAs offer little insight. Chandler's BAC transgenic experiment, examining a *Bmp4* reporter on a wildtype genetic background that should express full-length *Rubie*, suggests that the element regulating *Bmp4* expression in the inner ear acts in *cis* [17]. *Rubie* transcription might be necessary to open chromatin, in order to directly facilitate *Bmp4* expression or to relieve the effects of an insulator that would otherwise inhibit activity of an inner ear enhancer. We note that there are blocks of highly conserved sequence within *Rubie* introns that might contain DNA regulatory elements. The SWR ERV insertion does not interrupt these conserved sequences, but remains possible that transcription of *Rubie* is not required for *Bmp4* regulation (e.g., that a classical DNA enhancer element within the *Rubie* gene is perturbed by the ERV). Further studies will be necessary to determine the precise role of *Rubie* in *Bmp4* regulation.

Penetrance of the *Bmp4* heterozygote circling phenotype is incomplete and varies with genetic background [10], [11]. Vervoort and colleagues recently reported a chromosome 4 locus, in close proximity to the dominant *Ecl* locus, that modifies the circling phenotype of *Bmp4* heterozygous mice [11]. This finding indirectly supports the idea that *Rubie* transcription regulates *Bmp4* expression. While we have not identified the chromosome 4 *Ecl* gene, our results demonstrate that *Rubie* expression is disrupted in both CSS-14 circlers and non-circling SWR mice, suggesting that the chromosome 4 gene is likely not involved in regulation of *Rubie* expression or splicing. It remains to be determined whether the chromosome 4 *Ecl* gene influences BMP signaling or operates through a parallel pathway also necessary for canal morphogenesis [32].

## Materials and Methods

### Animal Care

#### Ethics Statement

Mouse breeding and experimentation was carried out in accordance with protocols approved by the Children's Hospital Boston and Duke University Institutional Animal Care and Use Committees (protocol A091-11-04), in accord with national and international guidelines. *Animals:* CSS-14 strain development was performed on a fee-for-service basis by Charles River Laboratories (CRL). All other mice were housed in the barrier facilities at Children's Hospital Boston or Duke University.

### CSS-14 Strain Development

CSS-14 was generated by marker-assisted backcrossing at CRL. Generations N2 through N5 were genotyped using a 99 marker genome-wide microsatellite panel, which included D14Mit126, D14Mit60, D14Mit157, and D14Mit228. At each generation, optimal breeders were selected from among all offspring carrying a non-recombinant SWR/J chromosome 14, and backcrossed to C57BL/10J. Additional backcrossing was carried out in our colony prior to experimentation. Genome-wide SNP genotyping was performed by The Mutation Mapping and Developmental Analysis Project at Brigham and Women's Hospital. Analysis of 455 informative SNPs confirmed that the CSS-14 genetic background was 100% C57BL/10J-derived and that the introgressed SWR/J chromosome 14 encompassed a minimal interval of 92.8 Mb, extending from dbSNP rs3710916 (8.8 Mb) to rs6179144 (101.6 Mb).

### CSS-14 Genotyping

Genomic DNA was prepared from tail biopsies using the QIAGEN DNeasy Blood & Tissue Kit according to the manufacturer's recommendations. SSLP markers found to be polymorphic between C57BL/10J and SWR/J (shown in Figure 2) were used for strain maintenance and positional cloning. Primer sequences of newly-developed SSLP markers, located between D14Mit129 and D14Mit60, are provided in Table S2. All markers were resolved by gel electrophoresis in 4% agarose.

### Paintfill Analysis of Inner Ears

E12.25 embryos and hemisected P0 heads were fixed overnight at 4°C in Bodian's Fixative (5% glacial acetic acid, 5% formalin, 75% ethanol, 15% water). Following fixation, samples were washed for 10 minutes in 100% ethanol, dehydrated overnight in 100% ethanol at room temperature, then cleared overnight at room temperature with methyl salicylate. White latex paint diluted to 0.025% in methyl salicylate was injected with a pulled glass pipette. Inner ear morphology was assessed using conventional light microscopy. In embryos, the precise stage of canal development was determined by analyzing the unaffected anterior and posterior canals, which develop earlier than the LSC [15].

### Rubie Sequence Analysis

RIKEN Mouse FANTOM clone F930028D17 was purchased through DNAFORM Clone Distribution and sequenced using standard T3 and T7 primers. *Rubie* exons were amplified from genomic DNA using the primers listed in Table S2, purified with the QIAGEN Gel Extraction Kit according to the manufacturer's instructions, and sequenced. Inbred strain genomic DNA samples were isolated from mice in our colony or purchased from The Jackson Laboratory.

### Rubie Expression Analysis

Total RNA was isolated from flash-frozen tissue using RNA STAT-60 (Tel-Test) according to the manufacturer's recommendations. Isolated RNA was treated with DNase I (Invitrogen) to remove contaminating genomic DNA, then phenol-chloroform extracted. Random hexamer-primed cDNA was synthesized using the SuperScript First-Strand Synthesis System for RT-PCR (Invitrogen). PCR was performed on the resulting cDNA using the *Rubie* and *Actb* primer pairs listed in Table S2. PCR products were resolved by agarose gel electrophoresis, purified with the QIAGEN Gel Extraction Kit, and sequenced.

### In situ Hybridization

Non-radioactive *in situ* hybridization was performed on 10–12 µm cryosections of E11.5 embryo heads. Detailed probe preparation and hybridization protocols can be found at http://goodrich.med.harvard.edu/protocols.htm. *Rubie* probe template was amplified from RIKEN clone F930028D17 using primers in exons 2 and 5 (Table S2). *Bmp4* probe was generated using template corresponding to the first 293 bp of exon 4.

### Rubie Insertion Mapping and Sequence Analysis

The ERV insertion in intron 1 of *Rubie* was mapped by PCR analysis of SWR/J and C57BL/10J genomic DNA. The PCR reactions depicted in Figure S2 were performed using the primers listed in Table S2. For each PCR reaction, amplification in SWR/J and C57BL/10J was evaluated as “present” or “absent” by agarose gel electrophoresis. Following mapping, long-range PCR was performed using the Expand Long Template PCR System (Roche) according to the manufacturer's instructions. PCR reactions were performed on inbred strain genomic DNA using primers Int1-H-F1 and Int1-MapR7 (Table S2). C57BL/10J and SWR/J bands were purified with the QIAGEN Gel Extraction Kit. C57BL/10J PCR products were sequenced directly. SWR/J PCR products were cloned into the pCR-XL-TOPO vector (Invitrogen) and sequenced by primer walking.

### Rubie Splicing Analysis

Hemi-nested PCR was performed on inner ear (P6) cDNA using nested forward primers in *Rubie* exon 1 and a reverse primer specific to ETnII-β endogenous retroviruses (Table S2). Inner nest PCR products were resolved by agarose gel electrophoresis, purified with the QIAGEN Gel Extraction Kit, and sequenced.

## Supporting Information

Figure S1 **Haplotype analysis of** ***Rubie*** **exon sequence.** *Rubie's* five exons were sequenced in SWR/J, C57BL/10J, and thirteen other mouse strains. The twelve single nucleotide polymorphisms (SNPs) that distinguish SWR/J and C57BL/10J are shown schematically (asterisks) and detailed in the haplotype chart. For each SNP, the SWR/J allele is depicted in blue and the C57BL/10J allele is shown in red. Gray boxes represent SNPs for which no sequence data is available.
(TIFF)

Figure S2 **Mapping and sequence analysis of the** ***Rubie*** **insertion.** The retroviral insertion in intron 1 of *Rubie* was mapped using a PCR-based strategy. Horizonatal bars representing individual PCR products are shown in relation to their location in intron 1, and are flanked by names of the primers used to generate them. Green bars represent products that were easily amplified by conventional PCR from both C57BL/10J and SWR/J genomic DNA. Red bars represent products that were robustly amplified in C57BL/10J, but could not be amplified by conventional PCR in SWR/J. The vertical gray bar depicts the region in which an insertion or chromosomal rearrangement must occur. The sequence of this 27 bp region is shown above (underlined). Long-range PCR between primers H-F1 and MapR7 revealed that the SWR/J allele contains a 5542 bp endogenous retrovirus (blue sequence), flanked by a 6 bp target site duplication (red sequence), a hallmark of retrotransposition.
(TIFF)

Table S1 Genomic Locations and Sequences of *Rubie* Exons.
(DOCX)

Table S2 Sequences of Oligonucleotide Primers.
(DOCX)
