# Supplementary figures and images for: Mutation of Rubie, a Novel Long Non-Coding RNA Located Upstream of Bmp4, Causes Vestibular Malformation in Mice
Source: PLoS One. 2012 Jan 12;7(1):e29495. doi: 10.1371/journal.pone.0029495 (PMC3257225; doi:10.1371/journal.pone.0029495)

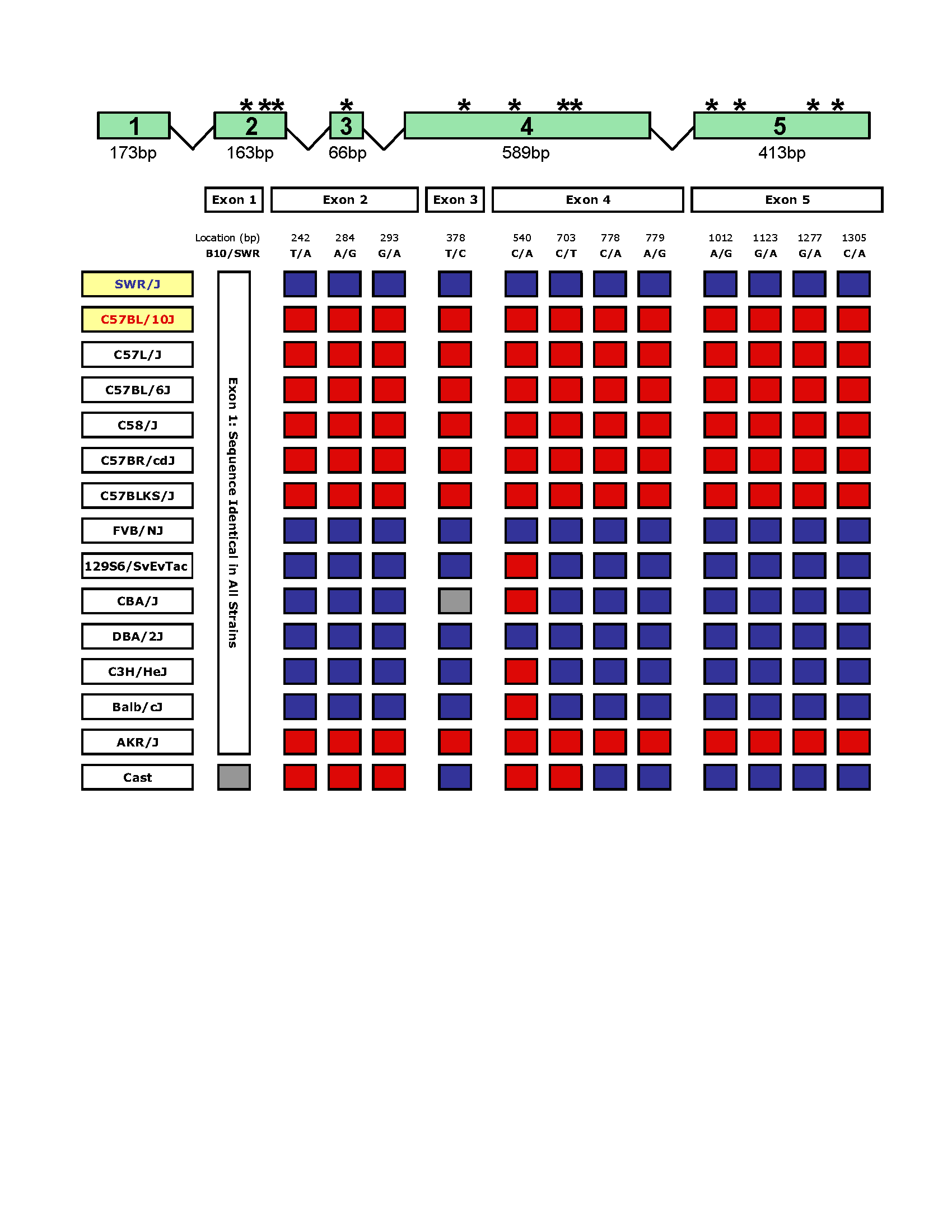

Supplement: Figure S1 — Haplotype analysis of Rubie exon sequence. Rubie's five exons were sequenced in SWR/J, C57BL/10J, and thirteen other mouse strains. The twelve single nucleotide polymorphisms (SNPs) that distinguish SWR/J and C57BL/10J are shown schematically (asterisks) and detailed in the haplotype chart. For each SNP, the SWR/J allele is depicted in blue and the C57BL/10J allele is shown in red. Gray boxes represent SNPs for which no sequence data is available. (TIFF) [file pone.0029495.s001.tiff]

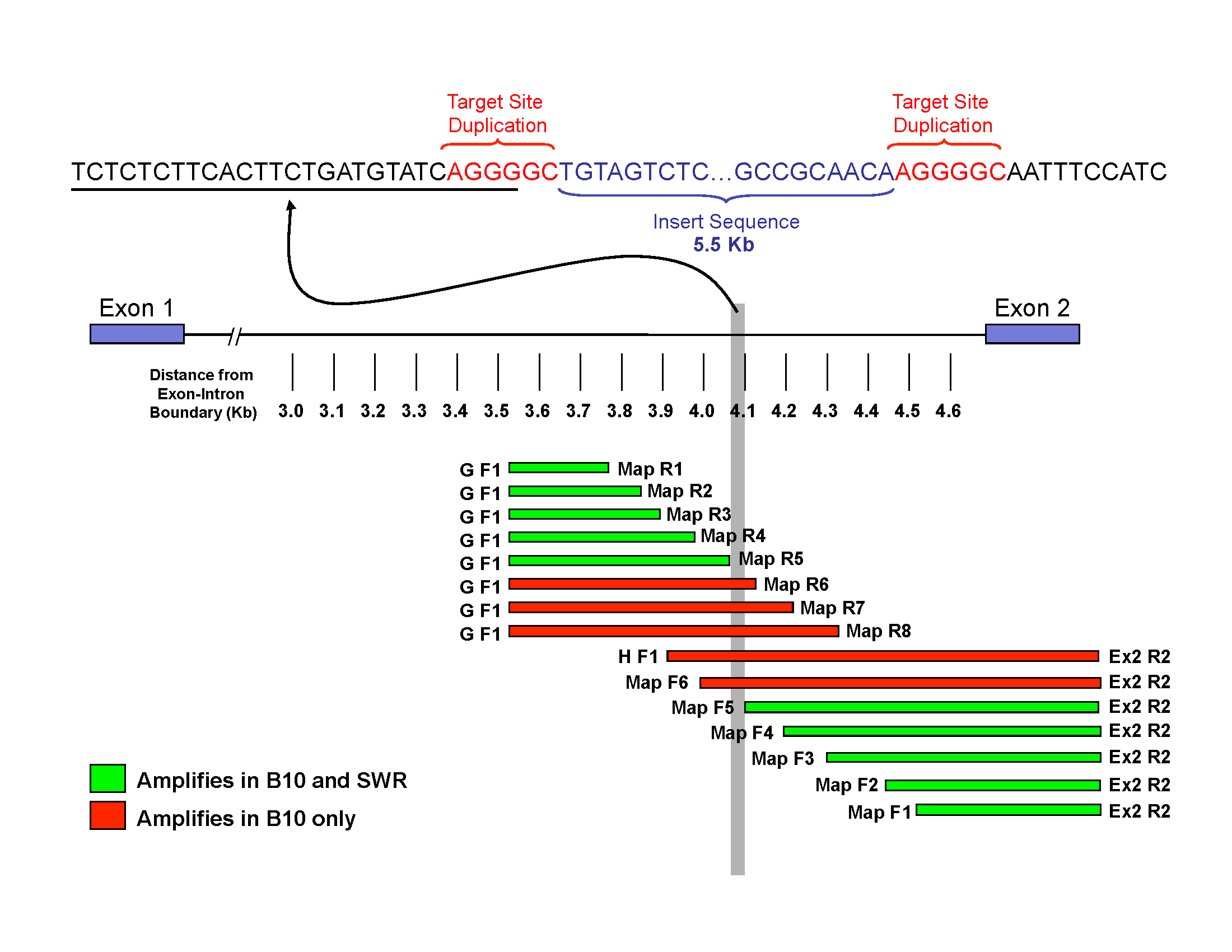

Supplement: Figure S2 — Mapping and sequence analysis of the Rubie insertion. The retroviral insertion in intron 1 of Rubie was mapped using a PCR-based strategy. Horizonatal bars representing individual PCR products are shown in relation to their location in intron 1, and are flanked by names of the primers used to generate them. Green bars represent products that were easily amplified by conventional PCR from both C57BL/10J and SWR/J genomic DNA. Red bars represent products that were robustly amplified in C57BL/10J, but could not be amplified by conventional PCR in SWR/J. The vertical gray bar depicts the region in which an insertion or chromosomal rearrangement must occur. The sequence of this 27 bp region is shown above (underlined). Long-range PCR between primers H-F1 and MapR7 revealed that the SWR/J allele contains a 5542 bp endogenous retrovirus (blue sequence), flanked by a 6 bp target site duplication (red sequence), a hallmark of retrotransposition. (TIFF) [file pone.0029495.s002.tiff]
