# Supplementary material for: Mutation of Rubie, a Novel Long Non-Coding RNA Located Upstream of Bmp4, Causes Vestibular Malformation in Mice
Source: PLoS One. 2012 Jan 12;7(1):e29495. doi: 10.1371/journal.pone.0029495 (PMC3257225; doi:10.1371/journal.pone.0029495)
Supplement: Table S1 — Genomic Locations and Sequences of Rubie Exons. (DOCX) [file pone.0029495.s003.docx]

**Supplemental Table 1. Genomic Locations and Sequences of *Rubie* Exons**

| **Exon** | **Length (bp)** | **Genomic Location**  **(July 2007 Assembly)** | **Sequence** |
| --- | --- | --- | --- |
| 1 | 173 | 14: 47195354 - 47195526 | GCTCGAGATTATGCCTCTTCCTTGTGTCGACTTCGCTTTCCATCTGTGAACTGGGAAAGGTGACCAGTAGGCTGCATGCTCTAAAGATGCTAAAGATGTAGCACATGCCCAGCCCCCATGGTCCCAGCCTGTCACCAGGCCATTCTGCTGCTACCAGAAACTTGCTGTTTAGG |
| 2 | 163 | 14: 47190762 - 47190924 | ACTTCCATCTGCAGGCAAGTGGGGCCATGGGATCTGCTAACATGCAGAAGGAAGATAGCAGCTCTTTGTGAACTCTCGGGCCTGCTGCACAATAAGCCAGAACAACACAGAGACAAGATGCTTTGGAACGTCTGGGAGTCTCTGCTGTCTTATTACATACAAG |
| 3 | 66 | 14: 47189878 - 47189943 | GACCCTGGCATTTCCTGCTAATTCAAAGGAAGAGAAGAAAATTAGTGCCTGTTCTTCATCATGTCT |
| 4 | 589 | 14: 47188852 - 47189440 | GTGAAACTGGAGCCAAAAATGGATTTTTTTTGGATCACACATCTCCTGTCTCCTGCTGCCGGTGATGAAGCTGCCAGCCCCACGCCAGTGGGCGGAAATAGATACCAGCCCTGGAGGAAGTACTGACTGCTTATGGGCGCCTGCCACCCTCCCTGCTTCCTCCTCTTCGTGTGGAAGGAAACCGTGGGCTTTCCTGGGCATTTTTTCTTATTCTCTTAATACAGGGGAACTGAAGCATTTGGCTTTGGTTGTTTGGATAGTGAATCTCAGCTTGGAGGGCTTTCCAGAAAGCAGGCGGCTCCGGTCTCTGAACTCTAACTCCGTTTCTCCACCCCCCTTCCGGTGCTGTGCAGTTCTTTCCAATTCCTGAATTGACATGTACAGTACTAGAAAACCTGCAGAGAGCGCCTGGAGGTCACAGCCTGCATGAGCTTTCAAGCTGGAGAGGGCTAAGTGGAAGGAAGACACTGCACCCCTGGGATTTCCTCCTGCAAATGAGATGAAATCTATATAAATGTACTCTTTCCCGTCTCATACCTTTTATTCGATTTGCATACCACGGATCCGATGACAACTCTCTTGAAAAGTG |
| 5 | 413 | 14: 47188006 - 47188418 | CTCCTTTGACTGTTTCAGCCACAGCGGTTCATGCAAAGAAACTAAATCTTGGAAAGGAAGGGGCTAACCCCTCCCCATGCTTGTGCTCAGGAACCACATGATGACCATGACAGACCATGACCAGGACATCGGTGGGTGCAAAAAGAAGAAACAAGAGCACAGGGAGGAAGGAAGAGCTGGGTGGCATGGGGTGGGGTGGGGTGGGGGGAGGATTGCAACTAGGCATGATCTTGCTTCAGGGCATGGAGCCCAGAAAGCCCAAGACCAGAGGGCTATGCCTAGAGCGAAGCATATTTTTCAGCAGTGCTCCCCCCATTTGGGGGACATATTCTAGGATGGAAGTAGAAAGATTGTAATGGAATGTGGCACGTGAGCTCATTTGTGAAAAACAAAACAAAATAAAACAAAAAACC |
