# Supplementary material for: Mutation of Rubie, a Novel Long Non-Coding RNA Located Upstream of Bmp4, Causes Vestibular Malformation in Mice
Source: PLoS One. 2012 Jan 12;7(1):e29495. doi: 10.1371/journal.pone.0029495 (PMC3257225; doi:10.1371/journal.pone.0029495)
Supplement: Table S2 — Sequences of Oligonucleotide Primers. (DOCX) [file pone.0029495.s004.docx]

**Supplemental Table 2. Sequences of Oligonucleotide Primers.**

|  | **Forward Primer (5’ 🡪 3’)** | **Reverse Primer (5’ 🡪 3’)** |  |
| --- | --- | --- | --- |
| ***New SSLP Markers Between D14Mit129 and D14Mit60*** | | | |
| D14Kar30 | CCTGGAGAACCACCAGAGAA | CCAATTACTATCAAACCTTTTTAGGC |  |
| D14Kar15 | TGTCATTTTGAGTTTGGAGTTGA | AAATGATTAGAGCTGCATCTTGC |  |
| D14Kar16 | CCCTCTCTCCACACACTCGT | TCCTGAAGGCTCTTGTTTGC |  |
| D14Kar35 | CGCTGTTTCATAACCATCGTC | TGGTGGGAAACCTTACTTCAA |  |
| D14Kar19 | AGGACCATCCCAATCCTACC | TGGCCCTTGGTACTTTTTGA |  |
| D14Kar24 | TCTGCTGCTTGCCTATCTCA | AATCAGGAACCAGCAGCAAG |  |
| D14Kar40 | GATGTTCTGCAGCCAATTCA | CCCAATGATGGGAAAGAAAG |  |
| ***Rubie Expression Analysis and In situ Hybridization*** | | | |
| *Rubie*-cDNA (Ex1-2) | CCTTGTGTCGACTTCGCTTT | ACAGCAGAGACTCCCAGACG |  |
| *Rubie*-cDNA (Ex1-4) | CCTTGTGTCGACTTCGCTTT | ACTTCCTCCAGGGCTGGTAT |  |
| *Rubie*-cDNA (Ex2-4) | AGCCAGAACAACACAGAGACAA | ACTTCCTCCAGGGCTGGTAT |  |
| *Actb*-cDNA | TTCTTTGCAGCTCCTTCGTTGCCG | TGGATGGCTACGTACATGGCTGGG |  |
| *Rubie*-probe | GAACGTCTGGGAGTCTCTGC | ACGTGCCACATTCCATTACA |  |
| ***Rubie Exon Sequencing*** | | | |
| *Rubie*-Ex1 | AGGCTCCTTGACCTCAACCT | GCATGCAGCCTGTCTATGAG |  |
| *Rubie*-Ex2 | ACCTTCTGCCCCTAGCTCAT | GCTTGCCTCTCCCCTAAAAC |  |
| *Rubie*-Ex3 | GTTTCTCCTGCAGGGTGCT | CCCCCTGCAATGAATAACTAA |  |
| *Rubie*-Ex4 | GCAAGATCCTTTTCCTGCTG | ATTCTCCGTGCAGTGGAAGT |  |
| *Rubie*-Ex5 | GGGAAGAAGGCCTTATGACTG | AGAGAGACCAGCGGTGAAGA |  |
| ***Rubie Intron 1 Insertion Mapping and Cloning*** | | | |
| Int1-G-F1 | CATCTTGATCACGAGGCAGA | -- |  |
| Int1-MapR1 | -- | TTTGTGCTGCTACCCCAAAT |  |
| Int1-MapR2 | -- | GCAACTGAGCCTCTACCTTCA |  |
| Int1-MapR3 | -- | GGTACGTACAGCCCATGCTC |  |
| Int1-MapR4 | -- | ATCCAGCAGCAACTGGCTAT |  |
| Int1-MapR5 | -- | AAGCCACCCACTGAATCAAC |  |
| Int1-MapR6 | -- | AACTTGTTTGGGAAGATGGAAA |  |
| Int1-MapR7 | -- | GAGGATGTATTCCTGACACTCAAA |  |
| Int1-MapR8 | -- | CCATTGTGTGTGGAAAACTTG |  |
| Int1-H-F1 | AGGGCAGGCTGTACTAAGCA | -- |  |
| Int1-MapF6 | TGCTGGATCATCACCTGAAA | -- |  |
| Int1-MapF5 | GCAATTTCCATCTTCCCAAA | -- |  |
| Int1-MapF4 | TTTGAGTGTCAGGAATACATCCTC | -- |  |
| Int1-MapF3 | GGATGGACTTCAAGTTTTCCA | -- |  |
| Int1-MapF2 | CTTCATTCCTGGGCTCAGTC | -- |  |
| Int1-MapF1 | CTGGAGGTTGCACTCCTGTT | -- |  |
| Ex2-R2 | -- | TGCCTGGATTTTCCTTTCAT |  |
| ***Analysis of Rubie Splicing to ERV*** | | | |
| Ex1-ERV-outer | CCTTGTGTCGACTTCGCTTT | ATCTCTCTGCCATTCTTCAGG |  |
| Ex1-ERV-inner | GAACTGGGAAAGGTGACCAGTA | ATCTCTCTGCCATTCTTCAGG |  |
